# Re-engineering the Cypriot General Healthcare System for Syndemics

**DOI:** 10.3389/fpubh.2022.734796

**Published:** 2022-07-11

**Authors:** Sarah Cuschieri, Amalia Hatziyianni, Peter Karayiannis, Juanita A. Haagsma, Grant M. A. Wyper, Marios Kantaris, Mamas Theodorou, Elena Pallari

**Affiliations:** ^1^Department of Anatomy, Faculty of Medicine and Surgery, University of Malta, Msida, Malta; ^2^Ammochostos General Hospital, Ammochostos, Cyprus; ^3^Medical School, University of Nicosia, Nicosia, Cyprus; ^4^Department of Public Health, Erasmus MC University Medical Center, Rotterdam, Netherlands; ^5^European Burden of Disease Network, European Cooperation in Science and Technology, Scotland, United Kingdom; ^6^Health Services Research Centre, Nicosia, Cyprus; ^7^Health Policy, Open University of Cyprus, Nicosia, Cyprus; ^8^Health Innovation Network, London, United Kingdom

**Keywords:** re-engineering, healthcare system, syndemic approach, non-communicable chronic disease (NCD), COVID-19

## Abstract

To date in Cyprus, there is no dedicated “Quality Improvement” body or Public Health authority. The long-awaited general healthcare system (known as GeSy or GHS) has been completed, mid-stream of the COVID-19 pandemic. A recently proposed resilience plan in response to the lessons learnt from the pandemic was put forward by the Government of the Republic of Cyprus to strengthen the capacity of the GHS and support public health defense. The negotiator of GeSy and Health Minister 2015–2018 also provided his view that the health system needs a holistic transformation of service provision. Recognizing failures and thinking from a syndemogenesis perspective how the envisioned patient-centric healthcare delivery can be achieved, we propose that the public health response could also be linked to a politico-economic one in shielding GeSy. We make such case for a syndemic strategy (simultaneous management of COVID-19 and pre-existing epidemics on the island) and the development of the five-district model where each main district hospital is to complement the activities of the GHS through developing: 1. A training Center for training and sharing of best practices for COVID-19 and other public emergencies. 2. A public health body. 3. A quality improvement institute. 4. A commissioning center on planning and streamlining healthcare services. 5. A clinical trial platform. The rationale is based on the management literature and use of existing resources and capabilities for transforming the GeSy and generating value.

## Introduction

To date in Cyprus, there is no dedicated “Quality Improvement” body or Public Health authority. When the novel coronavirus SARS-CoV-2 responsible for the respiratory disease known as COVID-19, hit the island, the Government response was quick in designating the Ammochostos General Hospital (AGH) as the reference hospital for COVID-19 cases (1). Another notable example of the immediate response included the set-up of a Scientific Advisory Committee (SAC) by the President of the Republic, comprised of experts in epidemiology, infectious diseases and virology, to provide advice to the Government (2). The decision-making process for handling COVID-19 on the island was very centralized at the level of the Council of Ministers and the President of the Republic. The decisions were mainly in the form of decrees and protocols issued by the Ministry of Health (MoH). These were then circulated, and the relevant information and guidelines disseminated by the MoH to the various authorities, governmental and/or non-governmental organizations. The action for the set-up of the SAC however, was a temporary decision, with plans to dismantle this as soon as the pandemic would shift to the endemic level. No further plans for the sustainment of the SAC or a similar function board were made, shifting back to a pre-pandemic public health strategy.

## The Cypriot Health-Policy Context

The MoH in Cyprus is the responsible body for the organization and the provision of equitable access and effective healthcare delivery through its hospitals and staff, with a mission to continuously improve population health. The Medical and Public Health Services (MPHS) Department of the MoH is responsible for health promotion and treatment of diseases encountered by the Cypriot population. The Health Monitoring Unit (HMU) has been developed to support health policy making, strategic planning, healthcare resources management, scientific research, and public health awareness. The pandemic has perhaps resurfaced the importance of such a Public Health body in response to health protection and prevention at the population level, in providing a more effective communication strategy to inform the public, combating fake news and dealing with other public health issues such as vaccination campaigns. However, no additional actions were made in setting-up and sustaining such a body. Further, mid-stream of the pandemic, the long-awaited general healthcare system (3–5) (known as GeSy or GHS) has been completed. This was done in two main phases as introduced by the Health Insurance Organization (HIO): phase one in June 2019 with the introduction of primary care and outpatient care and phase two in June 2020 with the introduction of inpatient care, as the foundation of universal care provision.

## Healthcare and Population Health in the Post COVID-19 Era

We recently calculated the impact of the mortality and morbidity burden from COVID-19 compared to non-communicable diseases and showed that Cyprus is at the very low end, compared to similar small state islands and big European countries (1, 6). Following the first wave, COVID-19 was not a syndemic on the island of Cyprus; for a short period of time, it was not a pandemic either. However, with the re-opening of schools in September 2020 and an increase of infection cases reported in nursing care homes, a higher second and an even worse third wave of cases occurred, leading to further lockdowns from November 2020 until May 2021. Delayed efforts for the re-introduction of masks in public places in August 2020, enforcement of non-pharmaceutical interventions and hygiene protocols, restricted citizen movement and others may be a result of the lack of a Public Health foundation. Overall, within 21 months of the COVID-19 epidemic in Cyprus, we witnessed less than 50 deaths until December 2020. Then despite the introduction of the publicly available vaccines against COVID-19 in Cyprus and a well-supported strategic public health plan put forward by the SAC and the MoH for the vaccination of the public, a soaring 550 deaths occurred between January to November 2021. The fact that 89% of deaths occurred during the second and third waves, demonstrates how important the public health prioritization on the island is; the majority of deaths occurred in patients with pre-existing conditions such as cancer, diabetes, and cardiovascular diseases, except for a few cases, as well as in unvaccinated people. Therefore, we make a case for the set-up of a Public Health foundation on the island and the use of a syndemic approach where the patient is given a holistic management approach i.e., simultaneous management of priority NCDs and COVID-19 (acute and post-acute).

## A Syndemic Strategy

Syndemics have been defined as the clustering of two or more diseases and the dynamic relationship between the biological and social elements that are at play (7) while the cumulative vs. multiplicative effects of such syndemics (between COVID-19 and NCDs) on the healthcare system strategy are yet to be explored (8). As context matters (9), recognizing failures and thinking of a syndemogenesis from a strategic perspective, achieving the envisioned patient-centric healthcare delivery could also be linked to a politico-economic response in shielding GeSy. A recently proposed resilience plan in response to the lessons learnt from the pandemic was put forward by the Government of the Republic of Cyprus (10) with 6% of the total budget (74.1 million euros) to be allocated on strengthening the capacity of the GHS and supporting public health protection. From this plan, the concept of dealing with syndemics, until now, is missing (11).

A key actor in the political context who had a catalytic role to the introduction of GeSy was the former Health Minister (2015–2018), George Pamboridis, who stated to us that “*the expected resilience of GeSy is to be formed through a bottom-up approach; there is an immense need for co-ordinating the rest of the healthcare services, as part of a transformative change to achieve its full potential. GeSy is at the core of this, but there are peripheral aspects that need to be addressed. The current proposed plans of the government are a great fuel for the healthcare system, but it is not the means to an end. Instead, we should be thinking about how we can create the appropriate conditions to achieve an equilibrium of the healthcare market based on demand and supply. The Cypriot population suffers from Covid-19, but this is an acute state; what about chronic non-contagious diseases? This remains the greatest challenge*” (12). Further, Pamboridis added “*the autonomy of the hospitals should be part of this equation. Such as introducing University hospitals; having an independent body to oversee commissioning of services; having an accreditation system in place*.” The case for re-engineering (13) based on eight pillars, was previously made, including the set-up of an independent body: the Cyprus Quality Improvement Institute. In this work, we make a case through the introduction of a conceptual model on how this can potentially be achieved in practice, following the literature on strategic management and leadership. Further research work however remains to test the exact parameters that this proposed model can be achieved and its potential impact on the health system.

## The Five-District Model and the Cypriot General Healthcare System

For the past 30 years, the estimated burden of disease, across all age and gender groups in Cyprus, remained stable for the following conditions: ischemic heart disease, cancer, low back pain and diabetes (type 2) (14). Based on the need to prioritize the management of patients with these chronic conditions, and shifting resources towards COVID-19 and other emergencies, we propose the development of the five-district model (FDM). FDM is to complement the activities of the new GHS, where a dedicated district and its main hospital will be responsible in collaboration with HIO for the following activies:

1. The development of a Center for training and sharing of best practices for COVID-19 and other public health emergencies.
2. The set-up of a public health body responsible for overseeing public health measures affecting the population.
3. The development of quality improvement practice, clinical practice guidelines and protocols.
4. The planning of healthcare services, resource allocation and co-ordination of efforts.
5. The set-up of a clinical trials platform in the design, conduct and analysis of clinical trials on new or re-purposed drugs.

Healthcare delivery is at the core of the system, where the syndemic strategy is introduced. Streamlining services and introducing telemedicine on a routine basis are some of such recommended process reconfigurations. Translating “know-hows” of the healthcare professionals into best practice guidelines for patient care are examples of such transformations targeting health system performance. Both virtual and physical infrastructure can be redesigned to allow patient access to the system and reduce disparities between patient groups. Efforts to develop a model to enhance patient care quality, speed up the uptake of innovations in practice and translate findings from research into healthcare delivery has been made through the development of the Integrated Delivery Systems test bed in the US healthcare system (15). Transferring this concept over to the Cypriot healthcare, part of the FDM solution is the securing of appropriate equipment and medical supplies, the increase in workforce capacity such as intensive care specialists, and the re-organization of teams and services to support the newly implemented GeSy and support the syndemic strategy, such as acute hospital admissions or exacerbation of syndemic interactions *e.g.*, psychological–biological interactions afflicted from COVID-19 on mental health. Finally, public health can be enhanced through communication strategies and a scientific advisory committee acting as external support mechanisms. The proposed FDM can be translated into fundamental pillars in any geographical setting (smaller and larger countries) to establish an evidence based syndemic approach. A limitation of our conceptual model is that it has not previously been tested and hence a transformation like this may take time for implementation. However, the strength of the proposed FDM is that it is based on an analysis of the existing healthcare landscape and the proposed reconfigurations are for existing resources as available within the Cypriot system, rather than acquisition of new ones.

**Figure 1:**
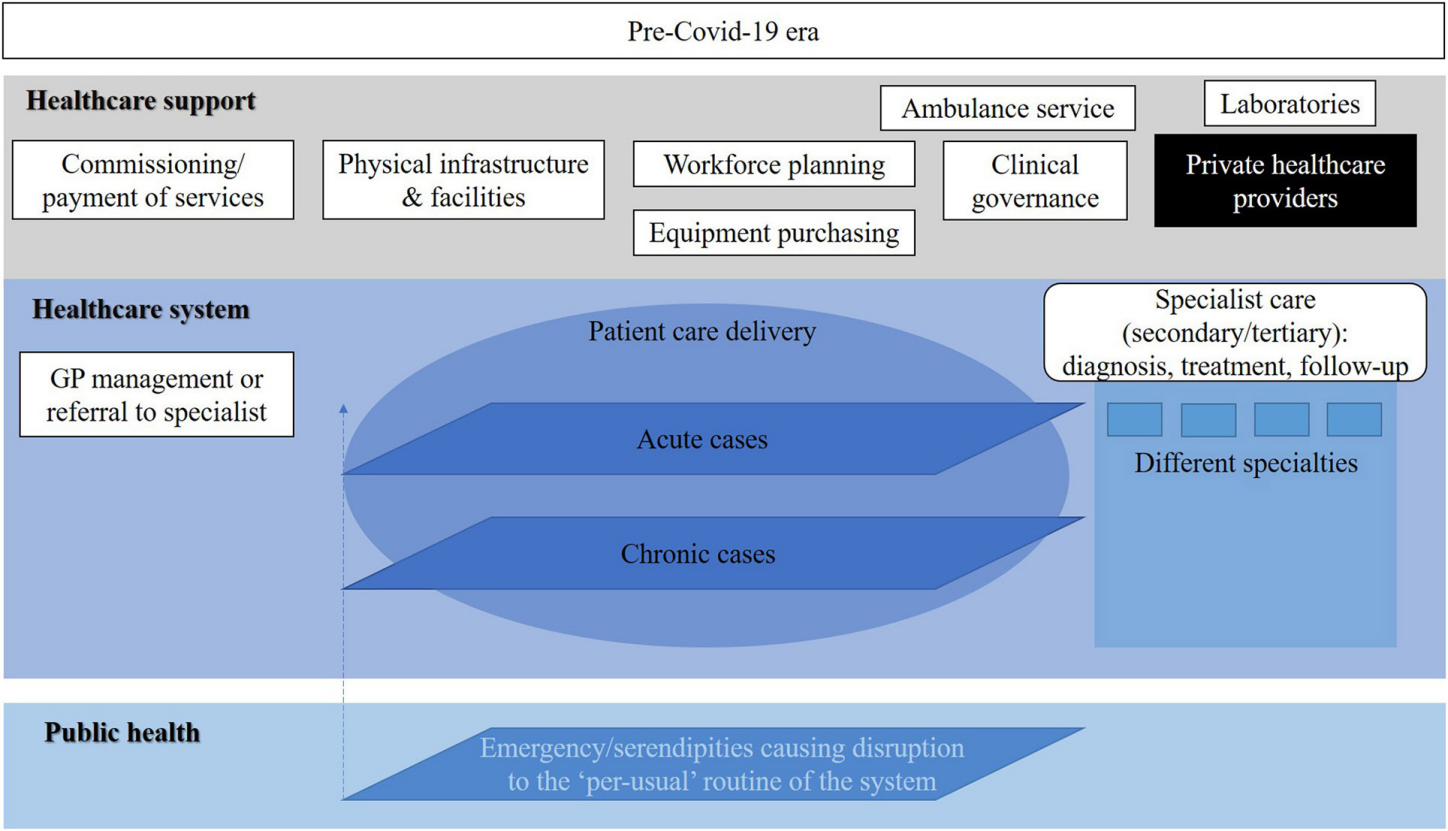
The pre-COVID-19 era Cypriot health service model.

**Figure 2:**
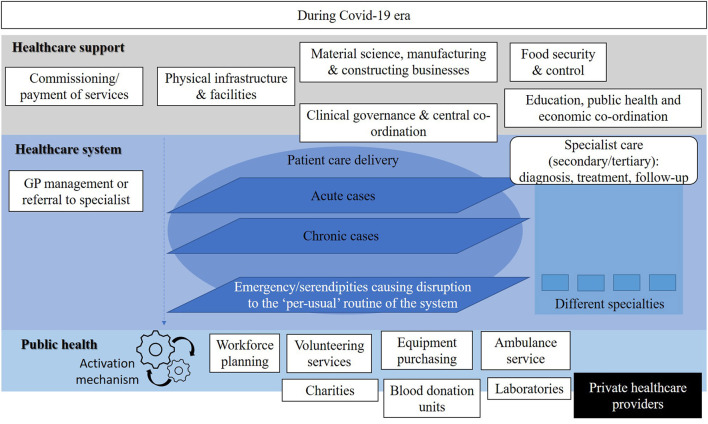
The ‘acute’ phase of the Cypriot healthcare system following the introduction of the COVID-19 pandemic.

**Figure 3:**
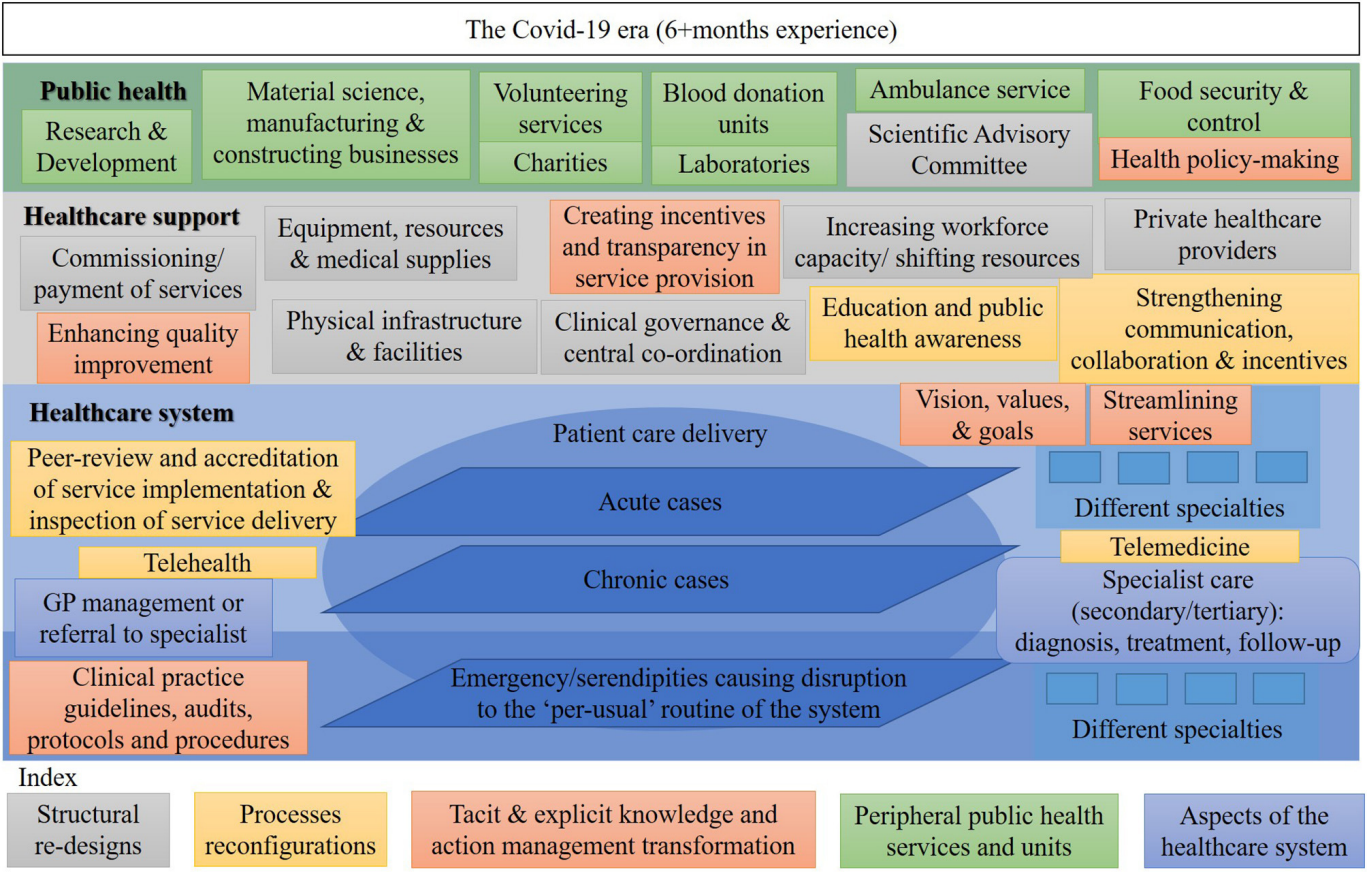
The shifted healthcare service model six+ months into the COVID-19 pandemic in Cyprus.

Figures 1–3 show the resources, workforce capacity, and service organization and planning at three time points. The healthcare system and healthcare support activities appear to be separated from public health priorities as shown in Figure 1. Then a slight integration occurs as shown in Figure 2 as a result of the pandemic. Further, we envision that by shifting the response from the acute and chronic cases (pre-COVID-19 model) in Figure 1 to the one in Figure 2 of COVID-19 cases (during COVID-19 early March to September) through a more co-ordinated and balanced effort, a third state of a healthcare system can be proposed. This proposal is shown in Figure 3 where the transition state model is to be translated into a re-engineered syndemic model. This is to be achieved by adequately shifting resources and redesigning aspects of the health system to support its sustainable performance, reducing inequalities and handling issues in care provision to achieve full population coverage of syndemic interactions.

**Figure 4:**
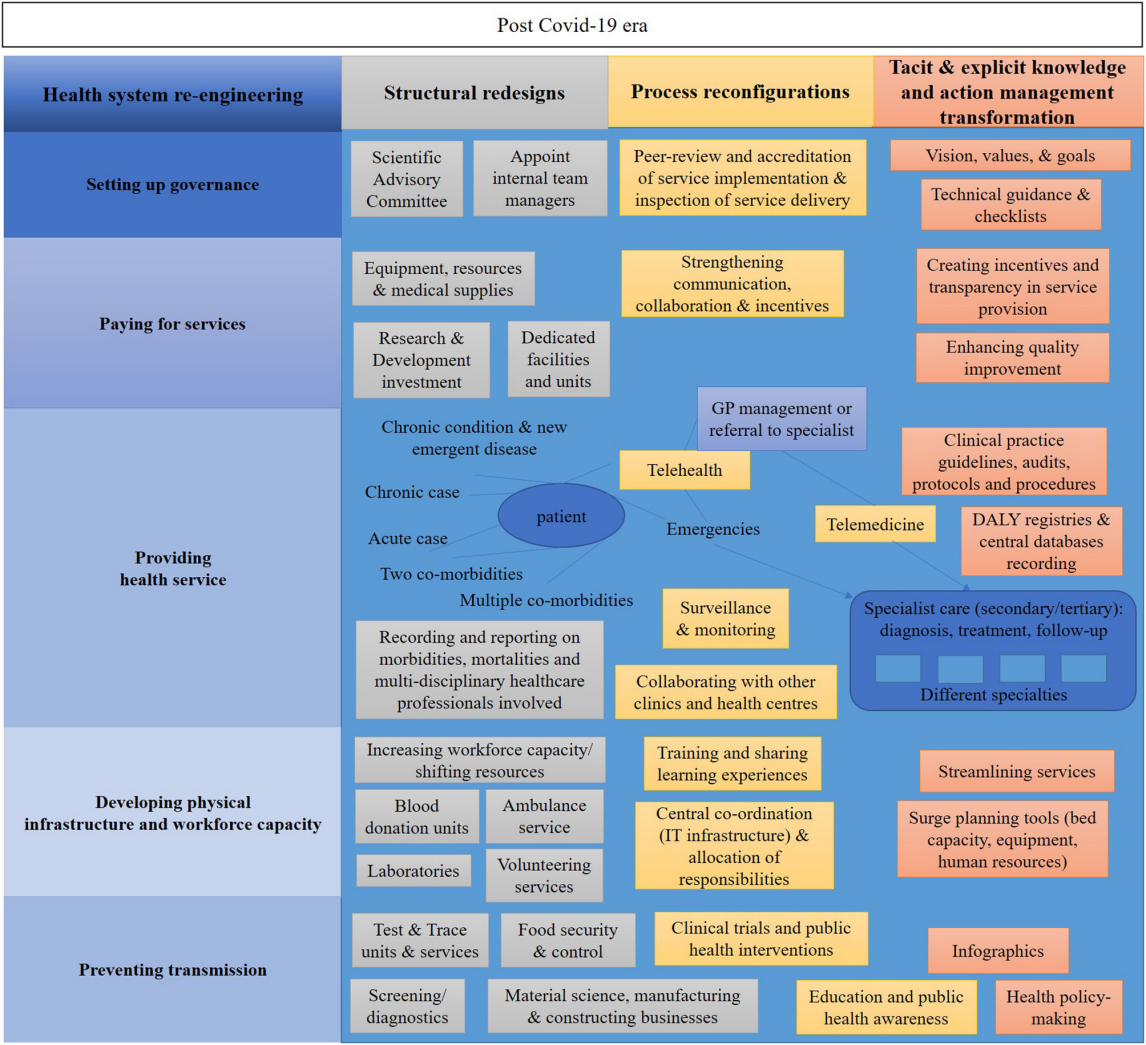
The proposed re-engineering health system set-up and organization in the post COVID-19 era.

Figure 4 maps the findings targeting the redesign of the existing health system in Cyprus through three dimensions: (i) process reconfigurations (yellow) (ii) structural redesigns (grey) and (iii) knowledge management transformations (orange).

## COVID-19 and the Cypriot Healthcare Landscape

There is an immense need for the Cypriot public health system to set up the five districts as priority areas. It is encouraged that the Cypriot government and other states with a similar population or geographical distribution to consider the transformation for public health emergency preparedness and transition to a working syndemic model. Specific research programmes like implementation research or quality improvement evaluation, as well as pilot research studies through focus groups with healthcare professional groups can commence to explore the impact of the proposed model upon the reconfiguration of the resources and delivery of the services within a syndemic concept. We applaud the Presidency of the Republic of Cyprus for the initiative and plan on strengthening the healthcare system and population health. We further call for the funding to be invested in such research in testing the FDM and remain optimistic that the above recommendations can support this effort.

## Data Availability Statement

The raw data supporting the conclusions of this article will be made available by the authors, without undue reservation.

## Author Contributions

SC and EP designed the study. AH provided the COVID-19 statistics. PK described the epidemiological situation. JH evaluated the FDM dimensions. GW analyzed the public health function of the GHS. MK and MT provided the political context of the response to the pandemic. All authors have contributed to the study methodology, validation, writing, editing, as well as reviewed the final submitted manuscript.

## Conflict of Interest

The authors declare that the research was conducted in the absence of any commercial or financial relationships that could be construed as a potential conflict of interest.

## Publisher's Note

All claims expressed in this article are solely those of the authors and do not necessarily represent those of their affiliated organizations, or those of the publisher, the editors and the reviewers. Any product that may be evaluated in this article, or claim that may be made by its manufacturer, is not guaranteed or endorsed by the publisher.
